# Efficacy and Safety of Tranexamic Acid in Traumatic Brain Injury: A Systematic Review and Meta-Analysis of Randomized Controlled Trials

**DOI:** 10.7759/cureus.73781

**Published:** 2024-11-15

**Authors:** Muhammad M Meer, Mahnoor Mumtaz, Zobia Farrukh, Basheer Ahmed

**Affiliations:** 1 Department of Acute Medicine, Northampton General Hospital, Northamptonshire, GBR; 2 Department of Emergency Medicine, Northampton General Hospital, Northamptonshire, GBR; 3 Department of General Medicine, Northampton General Hospital, Northamptonshire, GBR; 4 Department of Psychiatry, Palmer Community Hospital, South Tyneside, GBR

**Keywords:** acid tranexamic, antifibrinolytic agent, efficacy of tranexamic acid, intracranial hemorrage, meta-analysis, systemic review, thrombo-embolic events, traumatic brain injury

## Abstract

Traumatic brain injury (TBI) is a major global health concern, contributing significantly to mortality and long-term disability. Tranexamic acid (TXA), an antifibrinolytic agent, has demonstrated potential in reducing mortality in trauma patients, but its specific efficacy and safety in TBI management remain under investigation. This systematic review and meta-analysis aim to evaluate the efficacy and safety of TXA in patients with TBI by synthesizing data from randomized controlled trials (RCTs). A comprehensive literature search was conducted across PubMed, Scopus, Web of Science, and Cochrane CENTRAL databases about the studies conducted from January 2005 up to December 10, 2022. Eligible studies included RCTs involving TBI patients of any age, where the experimental group received TXA, and the control group received a placebo. The primary outcome was total mortality, focusing on the overall survival impact of the intervention.

Secondary outcomes included the need for neurosurgical intervention, pulmonary embolism, myocardial infarction, deep venous thrombosis (DVT), and stroke. Data were pooled using the DerSimonian-Laird random-effects model, with heterogeneity evaluated using the Cochrane Q test and I² statistic. Twelve RCTs encompassing 37,482 participants met the inclusion criteria. TXA administration was associated with a significant reduction in total mortality (relative risk (RR) 0.95, 95% confidence interval (CI) 0.90-0.99, P=0.002) compared to placebo, without increasing the risk of thromboembolic events such as DVT (RR 1.07, 95% CI 0.73-1.57, P=0.58) and pulmonary embolism (RR 0.97, 95% CI 0.78-1.22, P=0.82). The analysis showed no significant differences between the TXA and placebo groups concerning the need for neurosurgical intervention, incidence of myocardial infarction, or occurrence of stroke. Additionally, the studies demonstrated low to moderate heterogeneity across the assessed outcomes, indicating consistent findings regarding the treatment intervention and its associated complications. In conclusion, TXA significantly reduces total mortality in TBI patients without elevating the risk of thromboembolic complications. These findings support the integration of TXA into acute TBI management protocols, especially in settings requiring rapid intervention. Nevertheless, further research is necessary to optimize dosing regimens and administration timing and to assess the long-term functional outcomes associated with TXA use in TBI patients.

## Introduction and background

Traumatic brain injury (TBI) remains a significant global health concern, accounting for a substantial proportion of mortality and long-term disability worldwide [1]. The World Health Organization (WHO) estimates that TBI affects approximately 69 million individuals annually, with road traffic accidents, falls, and violence being the primary causes [2]. The economic burden of TBI is equally staggering, with direct and indirect costs estimated to exceed $76 billion annually in the United States alone [3].

The pathophysiology of TBI is complex and multifaceted, involving both primary and secondary injury mechanisms. While primary injury occurs at the moment of impact, secondary injury evolves over hours to days following the initial trauma. One of the critical components of secondary injury is intracranial hemorrhage, which can lead to increased intracranial pressure, cerebral ischemia, and neuronal death [4]. Approximately one-third of patients with TBI experience progressive hemorrhagic injury, which is associated with poor clinical outcomes and increased mortality [5].

In recent years, there has been growing interest in the use of tranexamic acid (TXA) as a potential therapeutic agent in TBI management. TXA is a synthetic lysine analogue that acts as an antifibrinolytic by competitively inhibiting the activation of plasminogen to plasmin. By reducing fibrinolysis, TXA helps maintain clot stability and potentially limits ongoing hemorrhage [6]. The drug has a well-established safety profile and has been used successfully in various clinical contexts, including trauma-related bleeding, postpartum hemorrhage, and elective surgery [7].

The potential application of TXA in TBI management was first highlighted by the CRASH-2 trial, which demonstrated reduced mortality in trauma patients with significant bleeding when TXA was administered within three hours of injury [8]. This finding prompted further investigation into the specific use of TXA in TBI patients. The landmark CRASH-3 trial, published in 2019, focused on the effects of TXA in TBI and suggested a mortality benefit, particularly in patients with mild to moderate head injury when treated early [9].

Despite these promising results, the role of TXA in TBI management remains a subject of debate. Concerns persist regarding its efficacy across different severities of TBI, the optimal timing and dosing of administration, and potential adverse effects, particularly thromboembolic events [10]. Furthermore, while mortality reduction is a crucial outcome, questions remain about TXA's impact on other clinically relevant endpoints, such as the need for neurosurgical intervention, functional outcomes, and quality-of-life measures [11].

This systematic review and meta-analysis aim to provide a thorough evaluation of the efficacy and safety of TXA in patients with TBI. By pooling data from all relevant randomized controlled trials, we seek to offer a more precise estimate of TXA's effects on mortality, the need for neurosurgical intervention, and the incidence of adverse events, including thromboembolic complications. Additionally, we aim to explore potential sources of heterogeneity in treatment effects, such as injury severity, timing of TXA administration, and dosing regimens.

## Review

Methods

Study Design and Reporting Standards

This systematic review and meta-analysis were meticulously conducted in accordance with the Preferred Reporting Items for Systematic Reviews and Meta-Analyses (PRISMA) guidelines [12]. The methodological framework adhered strictly to the procedures outlined in the Cochrane Handbook for Systematic Reviews of Interventions (Version 5.1.0), ensuring rigor and reproducibility in the synthesis of evidence.

Eligibility Criteria

Studies were included in this analysis based on predefined criteria. The population encompassed participants of all ages diagnosed with traumatic brain injury (TBI), with no restrictions regarding age, gender, or severity of injury. The intervention required that the experimental group received tranexamic acid (TXA), regardless of dosage regimen or administration route. The comparator was limited to studies where the control group received a placebo, excluding those that compared TXA to other active treatments. The primary outcome assessed was total mortality, reflecting the direct impact of the intervention on survival. Secondary outcomes included the need for neurosurgical intervention, an indicator of treatment efficacy in reducing surgical necessity. Additionally, TXA-associated complications were evaluated, specifically pulmonary embolism, myocardial infarction, deep venous thrombosis (DVT), and stroke. Only randomized controlled trials (RCTs) conducted from January 2005 up to December 10, 2022, were considered to ensure the inclusion of high-quality evidence. Studies were excluded if they were not published in English, were observational in nature, involved animal subjects, or were conference abstracts, editorials, letters, and other non-peer-reviewed publications.

Literature Search Strategy

A comprehensive and systematic literature search was performed across four major electronic databases: PubMed, Scopus, Web of Science, and the Cochrane Central Register of Controlled Trials (Cochrane CENTRAL). The search was conducted from January 1, 2023, to January 5, 2023, focusing on randomized controlled trials (RCTs) carried out from January 2005 to December 10, 2022. using the following query: ("Tranexamic acid" OR "TXA" OR "AMCHA" OR "t-AMCHA" OR "AMCA" OR "Anvitoff" OR "Cyklokapron" OR "Spotof" OR "Transamin") AND ("Traumatic brain injury" OR "TBI" OR "Trauma*" OR "Encephalopath*") All retrieved records were imported into reference management software (Zotero) to remove duplicates. Subsequently, the reference lists of all included studies were manually reviewed to identify any additional eligible studies not captured in the initial search.

Study Selection

**Figure 1:**
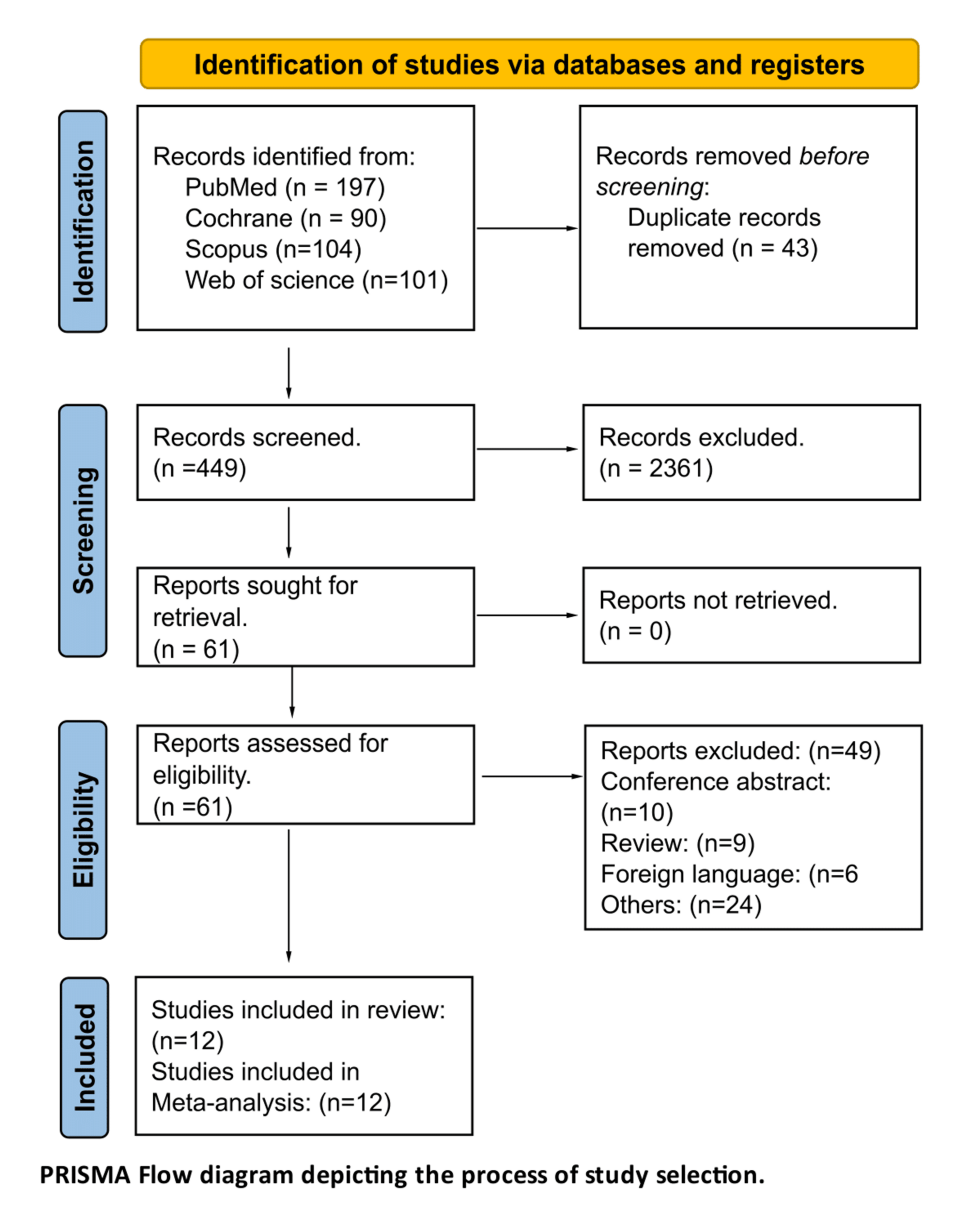
The process of study selection References [13-25].

The selection of studies followed a two-step screening process. Initially, two independent reviewers screened the titles and abstracts of all retrieved articles to assess their relevance based on the eligibility criteria. Articles were categorized as "include," "exclude," or "possibly include" for further review. In the second step, full-text versions of articles deemed potentially eligible were obtained and thoroughly evaluated against the inclusion and exclusion criteria. Any discrepancies between reviewers during both screening phases were resolved through discussion and, if necessary, by consulting a third reviewer. The entire study selection process is visually summarized in the PRISMA flow diagram (Figure 1).

Data Extraction

Data extraction was performed using a standardized and comprehensive form to ensure consistency and accuracy. Two reviewers independently extracted data from each included study to minimize errors and bias. The extracted data encompassed various aspects, including study characteristics (such as authors, year of publication, country, study design, and methodology), population characteristics (including sample size, age range, mean age, gender distribution, severity and type of TBI, baseline health status, and comorbidities), intervention details (dosage and regimen of TXA administration, route of administration, and duration of treatment), comparator details (type and dosage of placebo used), and outcome measures (the primary outcome was total mortality, focusing on the overall survival impact of the intervention. Secondary outcomes included the need for neurosurgical intervention, pulmonary embolism, myocardial infarction, deep venous thrombosis (DVT), and stroke). Additionally, information relevant to each domain of the Cochrane Risk of Bias tool was extracted. Any disagreements in data extraction were resolved through discussion and, if necessary, by involving a third reviewer.

Quality Assessment

The methodological quality and risk of bias of the included RCTs were independently assessed by two reviewers using the Cochrane Risk of Bias 2 (RoB 2) tool. The assessment covered five key domains: the randomization process (including the adequacy of random sequence generation and allocation concealment mechanisms), deviation from intended interventions (including the blinding of participants and personnel and adherence to the intervention protocol), outcome measurement (including the blinding of outcome assessors and the objectivity of outcome measures), missing outcome data (including the completeness of outcome data and handling of missing data), and the selection of reported results (including the pre-registration of study protocols and consistency between reported outcomes and pre-specified outcomes). Each domain was rated as having a low risk of bias, a high risk of bias, or some concerns. Any discrepancies between the two reviewers were resolved through discussion, and if consensus was not reached, a third assessor was consulted to finalize the rating.

Data Synthesis and Statistical Analysis

Data synthesis was conducted using the DerSimonian-Laird random-effects model to account for variability both within and between studies. All statistical analyses were performed using StataMP version 17 for Windows. For outcomes reported on a continuous scale, the mean difference (MD) between the TXA and placebo groups was calculated along with the corresponding standard deviation (SD), and these MDs were pooled using the random-effects model to derive a combined estimate. For binary outcomes, the relative risk (RR) was calculated based on the frequency of events and the total number of patients in each group, and these RRs were also pooled using the random-effects model to obtain a summary measure of effect. In studies reporting outcomes at multiple time points, the last available endpoint was selected for the primary analysis to maintain consistency across studies.

Heterogeneity Assessment

Assessing heterogeneity was crucial to determine the consistency of the study results. The Cochrane Q Test (Chi-square Test) was employed to evaluate the presence of statistically significant heterogeneity among the included studies, with a P-value < 0.1 considered indicative of significant heterogeneity. Additionally, the I² statistic was calculated to quantify the proportion of total variation across studies attributable to heterogeneity rather than chance, using the formula:

I^2^=Q(Q−df)​×100

Where Q is Cochran’s Q statistic (calculated as the sum of the squared differences between each study’s effect estimate and the overall pooled effect estimate, weighted by the inverse of their variance), df is the degrees of freedom (the number of studies minus one).

Interpretation of the I² values was as follows: 0-40% might not be important, 30-60% may represent moderate heterogeneity, 50-90% may indicate substantial heterogeneity, and 75-100% suggests considerable heterogeneity. An I² value ≥ 50% was considered indicative of high heterogeneity. If significant heterogeneity was detected, potential sources were explored through subgroup analyses or sensitivity analyses, although specific strategies were not detailed in the initial protocol.

Risk of Bias Across Studies

In addition to assessing the risk of bias within individual studies, it was essential to evaluate the possibility of publication bias and other biases across studies. Funnel plots were visually inspected for key outcomes to detect asymmetry, which may suggest publication bias. Egger’s Test was conducted to statistically assess funnel plot asymmetry and quantify potential publication bias. Furthermore, sensitivity analyses were performed to evaluate the robustness of the meta-analysis results by excluding studies with a high risk of bias or by employing different statistical models. The risk of bias assessments across studies were independently conducted by two authors, with any discrepancies resolved through discussion and, if necessary, consultation with a third author to reach a consensus.

Results

Literature Search and Study Selection

A comprehensive literature search yielded 492 records pertinent to the study topic. After an initial screening of titles and abstracts, 61 articles were identified as eligible for full-text review. Upon thorough evaluation, 12 studies met all inclusion criteria and were incorporated into the meta-analysis. Additionally, a manual examination of the reference lists from the included studies was conducted; however, this process did not identify any further eligible articles. The detailed study selection process is depicted in the PRISMA flow diagram (Figure 1).

Study Characteristics

All included studies were randomized controlled trials (RCTs) focusing on patients diagnosed with traumatic brain injury (TBI), collectively enrolling 37,482 participants. The populations across these studies were homogeneous, ensuring consistency in patient demographics and clinical characteristics. Key attributes of the included studies, such as sample size, intervention details, and outcome measures, are summarized in Table 1. Additionally, Table 2 provides an overview of the baseline characteristics of the study populations, including age distribution, severity of TBI, and other relevant clinical parameters.

**Table 1:** Key attributes of the included studies, such as sample size, intervention details, and outcome measures

Study ID	Country	Study design	N	From	To	TXA dose	Main findings
Yutthakasemsunt et al., 2013 [21]	Thailand	RCT	238	23rd October 2008	14th August 2009	1.0 gram over 30 minutes followed by a maintenance dose of 1.0 gram infused over eight hours	Progressive intracranial hemorrhage, the risk of death and the risk of unfavorable outcome on the Glasgow Outcome Scale did not reach significant difference between two groups.
Roberts et al., 2021 [18]	International	RCT	12639	20 July 2012	31 January 2019	1 g over 10 minutes and then infusion of 1 g over 8 hours	The risk of Head injury death was not statistically significant among patients treated within 3 hours The TXA group showed significant reduction of the head injury death risk in patients with mild to moderate head injury The risk of disability, vascular occlusive events and seizures did not reach significant difference between TXA and control group
Roberts et al., 2013 [17]	International	RCT	20,21	May 2005	January 2010	(Loading dose 1 g over 10 minutes then infusion of 1 g over 8 hours)	There was a statistically significant reduction in all-cause mortality for the TXA group (p=0.0035). TXA group showed significant reduction in the risk of death due to bleeding (≤ 1 hour from injury, p<0.0001) and between (1 to 3 hours, p=0.03). after 3 hours of treatment given, TXA group showed significant increase in the risk of death due to bleeding compared to placebo group (p=0.004). The effect of TXA on death due to bleeding varied by systolic blood pressure, Glasgow Coma Scale score or type of injury was not recorded.
Safari et al., 2021 [19]	Iran	RCT	94	N/A	N/A	1-gram TXA within the first 3 hours of admission, followed by 1 gram every 6 hours for 48 hours	Hematoma diameter at 24 hours and 48 hours showed significant increase in length (p=0.04), height (p=0.04), and volume (p=0.01) for the TXA group Midline Shift and Level of Consciousness did not reach a significant difference between two groups (p>0.05)
Mojallal et al., 2020 [16]	Iran	RCT	100	June 2014	June 2015	500 mg 5 cc boluses	There was a statistically significant decrease of the ICU stay for TXA group compared to control group (p=0.001). The rate of hemorrhage volume progress (p=0.824) and the mortality rate of patients during the first 7 days of hospitalization (p=0.236) were not statistically significant between two groups
Sprigg et al., 2019 [20]	International	RCT	2325	2013	2017	1 g of an intravenous tranexamic acid bolus followed by an 8-hour 1g infusion	Modified Rankin scale did not reach significant difference between two groups (p=0.11). Neurological impairment did not provide significant difference between two groups (p = 0.10) Functional outcomes, activities of daily living, mood, cognition, or quality of life, length of hospital stay and discharge, the number of deaths, and Survival did show significant differences between the two groups. The number of deaths by day 90 did not reach significant difference between two groups (p= 0.37).
Meretoja et al., 2020 [23]	Australia	RCT	100	March 1, 2013,	Aug 13, 2019	1 g of intravenous tranexamic acid over 10 min followed by 1 g over 8 h	The risk of intracerebral hemorrhage growth did not reach significant difference between two groups (p=0·41) The mortality rate within 90 days (p= 0·19) and the thromboembolic complications (p= 0·57) were not statistically significant between two groups.
Fakharian et al., 2018 [14]	Kashan	RCTs	156	2014	2016	1 g in 100 mL of normal saline in 10 minutes and then with a maintenance dose of 1 gram per 1000 mL of normal saline for 8 hours.	The incidence of hemorrhagic lesion growth was not a statistically significant difference between the two groups (p=0.87). Hemorrhagic lesion growth was not statistically significant difference between two group (p=0.27). The frequency of death was not statistically significant difference between two group (p=1). The adverse outcome at discharge (p=0.25) and three months later (p=0.12) did not show significant difference between two groups. No side effect was observed with the TXA group
Chakroun-Walha et al., 2019 [13]	Tunisia	RCTs	180	1st August 2016	30th September 2017	of 1 g in 100 mL of normal saline in 10 min and then with a maintenance dose of 1 g per 500 mL of normal saline for 8 h	The needs of transfusion or neurosurgery (p=0.4), the mortality rate (p=0.4), the in-hospital length of stay (p=0.8) and the dependency at 28-post-traumatic day were comparable between two groups. Pulmonary embolism showed significant difference in the TXA group (p=0.02)
Mousavinejd et al., 2020 [24]	Iran	double-blind, randomized, and placebo controlled trial	40	2017	2018	1g TAX with 500 ml of 0.09% normal saline and intravenous Infusion	Hemorrhage during surgery was not statistically significant difference between two group (p=0.83). Hemoglobin volume reduction did not reach significant difference between two groups.
Jokar et al., 2017 [15]	Arak, Iran	RCTs	80	2014.		1 g in 100 ml 0.9% NaCl over 10 min followed by a continuous infusion of 1 g in 500 ml 0.9% NaCl over 8 h	There was a significant decrease in the hemorrhage volume for the TXA group after 48 hours (p=0.04). The mean total hemorrhage expansion produced a significant decrease in the TXA group (p<0.001)
Rowell et al., 2020 [22]	North American, USA and Canada	Multicenter, double-blinded, RCT	621	May 2015	March 2017	1g tranexamic acid bolus in the out-of-hospital. 1g tranexamic acid IV infusion initi- ated upon hospital arrival. 2g tranexamic acid bolus in the out-of-hospital.	6-month neurologic outcome as measured by the Glasgow Outcome Scale did not reach a statistical significant difference between two groups. There was no significant difference between two groups in the 28-day mortality (P = 0.26), 6-month Disability Rating Scale score (P = 0.29), or progression of intracranial hemorrhage (P = 0.16).
Anderson et al., 2020 [25]	United States and Canada.	RCT	287	May 2015	March 2017	2 g	TXA provided a significant decrease than the control group in Levels of syndecan-1 and levels of angiopoietin-2. Remaining markers did not reach a significant difference between two groups.

**Table 2:** An overview of the baseline characteristics of the study populations, including age distribution, severity of TBI, and other relevant clinical parameters. TXA: Tranexamic acid; TBI: Traumatic brain injury, NR: Not recorded.

Study ID	Study arms	Number of patients	Age, years, Mean (SD)	Male, N	Systolic blood pressure, Mean (SD)	Glasgow Coma Score (GCS), Mean (SD)	Mild GCS (13-15), N	Moderate GCS (9-12), N	Severe GCS (3-8), N
										Roberts et al., 2021 [18]	TXA	6406	43 (19.8)	5104	NR	NR	2149	1944	NR
											No TXA	6331	43.1 (19.7)	5104	NR	NR	2093	1929	2284
Yutthakasemsunt et al., 2013 [21]	TXA	120	34.8 (16)	103	NR	NR	NR	52	NR
	No TXA	118	34.1 (15.3)	107	NR	NR	NR	47	68
Fakharian et al., 2017 [14]	TXA	78	42.3 (18.3)	67	118.2 (16.3)	12.7 (2.83)	NR	NR	NR
	No TXA	78	39.3 (18.1)	66	120 (15.6)	11.65 (3.71)	NR	NR	NR
Safari et al., 2021 [19]	TXA	47	36.2 (15.1)	40	NR	11.1 (2.9)	NR	NR	NR
	No TXA	47	36.4 (14.1)	38	NR	11.1 (3)	NR	NR	NR
Chakroun-Walha et al., 2019 [13]	TXA	96	44 (20)	11	87.1 (16.5)	9 (5)	NR	NR	NR
	No TXA	84	39 (18)	8	88.8 (16.8)	10 (5)	NR	NR	NR
Mousavinejad et al., 2020 [24]	TXA	20	54.89 (19.14)	14	NR	NR	1	5	13
	No TXA	20	55.16 (18.15)	12	NR	NR	2	3	14
Jokar et al., 2017 [15]	TXA	40	35.4 (14.6)	32	160.1 (21.1)	NR	NR	NR	NR
	No TXA	40	36.2 (14.9)	28	161.9 (18.1)	NR	NR	NR	NR
Rowell et al., 2020 [22]	TXA	312	39 (26.57)	227	NR	7.8 (3.3)	14	129	169
	No TXA	309	36 (25.55)	233	NR	7.6 (3.2)	8	115	186
Sprigg et al., 2019 [20]	TXA	1161	69.1 (13.7)	642	171.7 (27.5)	13.4 (2.2)	NR	NR	NR
	No TXA	1161	68.7 (13.9)	659	173.5 (26.8)	13.5 (2.1)	NR	NR	NR
Mojallal et al., 2020 [16]	TXA	56	41.15 (20.27)	40	NR	NR	32	15	9
	No TXA	44	37.4 (19.6)	40	NR	NR	28	6	10
Roberts et al., 2013 [17]	TXA	10093	34.6 (14.1)	8439	NR	NR	6934	1353	1799
	No TXA	10114	34.5 (14.4)	8496	NR	NR	6908	1351	1839
Meretoja et al., 2020 [23]	TXA	50	73 (55.8)	35	168 (25)	14 (11)	NR	NR	NR
	No TXA	50	71 (58.79)	27	173 (25)	15 (13)	NR	NR	NR

Risk of Bias Assessment

**Figure 2:**
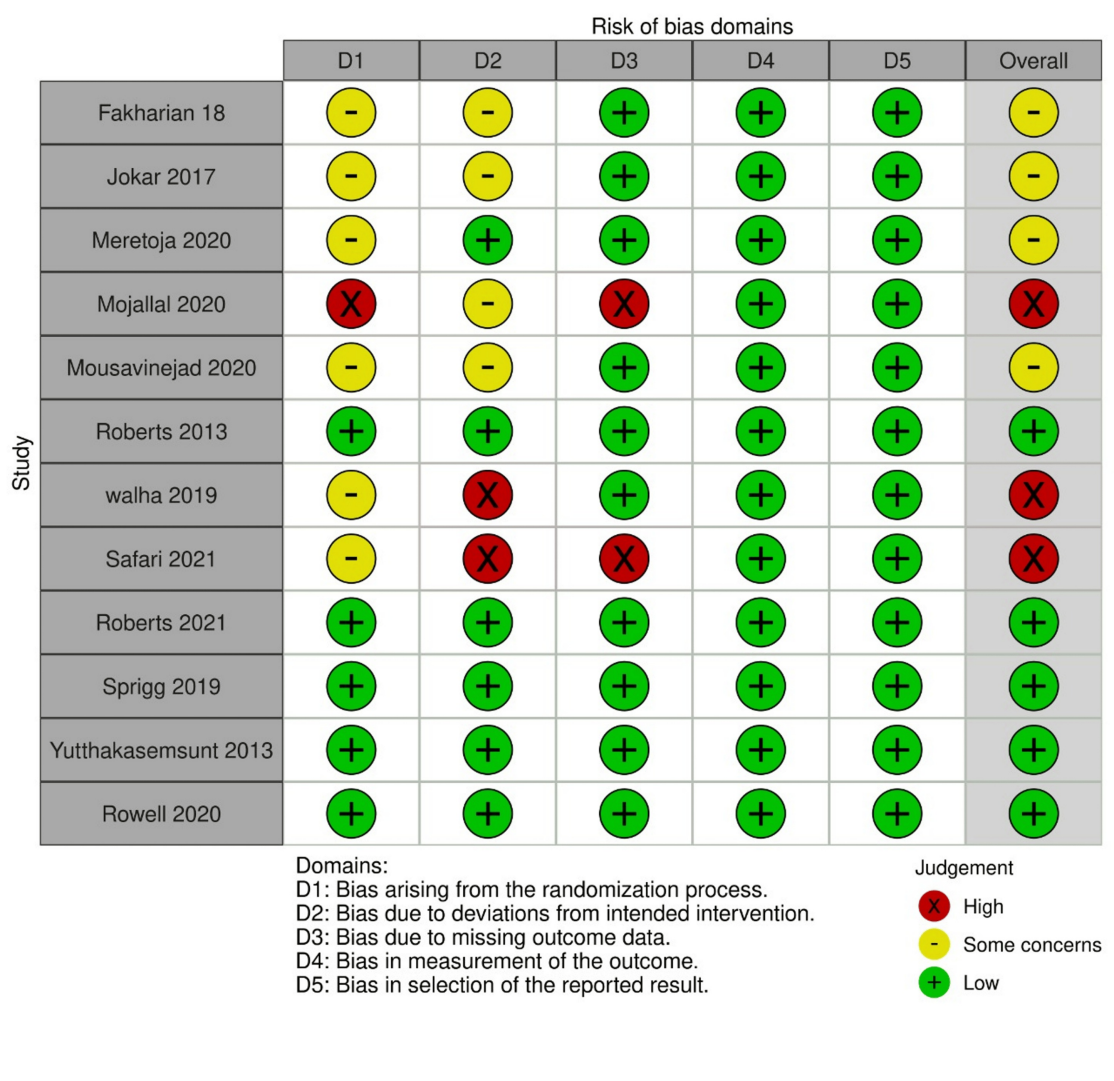
A summary of the risk of bias across all studies References [13-25].

**Figure 3:**
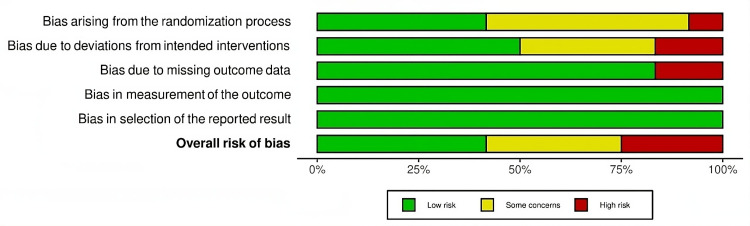
Overall risk of bias across all studies

The methodological quality of the included studies was assessed using the Cochrane Risk of Bias Assessment Tool. The evaluation indicated that the studies ranged from moderate to high quality, minimizing concerns about internal validity. A summary of the risk of bias across all studies is presented in Figures 2-3.

Primary and secondary outcomes

Total Mortality

**Figure 4:**
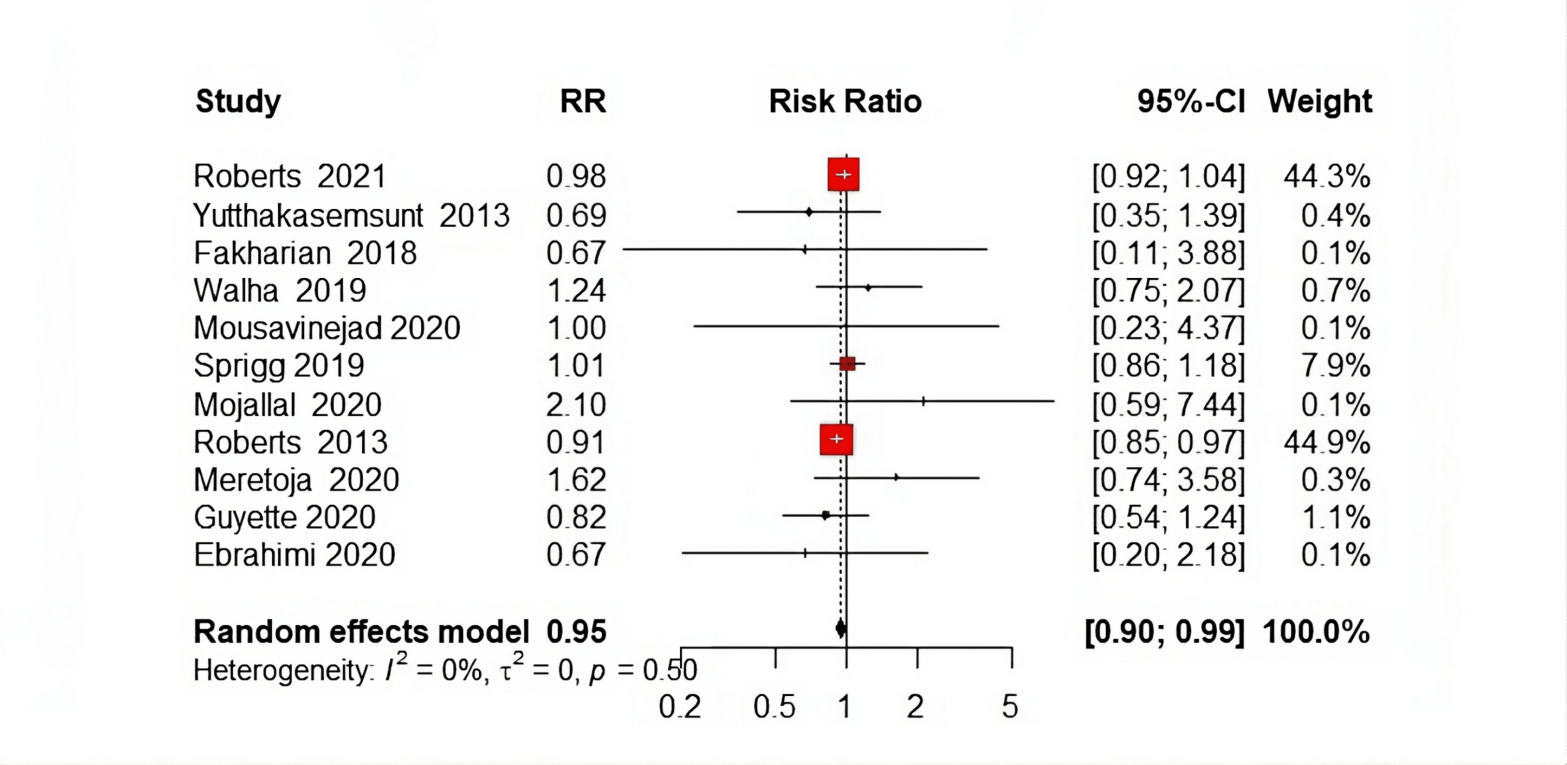
Forest plot of total mortality References [13,14,16-18,20,21,23,24].

The meta-analysis demonstrated that the tranexamic acid (TXA) group experienced a significantly lower total mortality rate compared to the placebo group. The pooled relative risk (RR) was 0.95 (95% Confidence Interval (CI): 0.90 to 0.99, P=0.002), indicating a 5% reduction in mortality associated with TXA administration (Figure 4). Statistical heterogeneity was assessed and found to be non-significant (P=0.50; I²=0%), suggesting consistent results across the included studies.

Need for Neurosurgery

**Figure 5:**
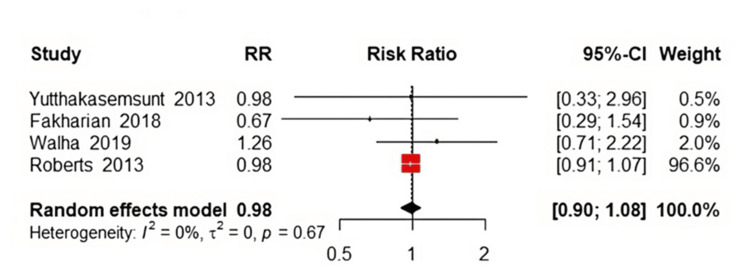
Forest plot of need for neurosurgery References [13,14,17,21].

Assessment of the requirement for neurosurgical intervention revealed no significant difference between the TXA and placebo groups. The pooled RR was 0.98 (95% CI: 0.90 to 1.08, P=0.68, Figure 5), indicating that TXA does not substantially influence the necessity for neurosurgery. The analysis showed homogeneity among the studies (P=0.67; I²=0%).

Deep Venous Thrombosis (DVT)

**Figure 6:**
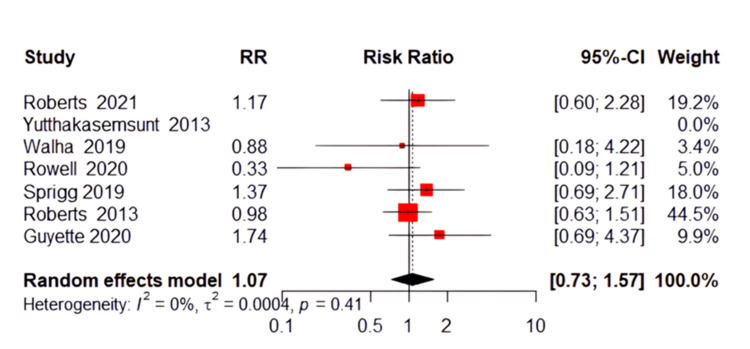
Forest plot of deep venous thrombosis References [13,16-18,20-22].

The incidence of deep venous thrombosis events was analyzed, showing no significant advantage of TXA over placebo. The pooled RR was 1.07 (95% CI: 0.73 to 1.57, P=0.58, Figure 6), suggesting a non-significant increase in DVT risk with TXA use. The studies included in this outcome were homogeneous (P=0.41; I²=0%).

Pulmonary Embolism (PE)

**Figure 7:**
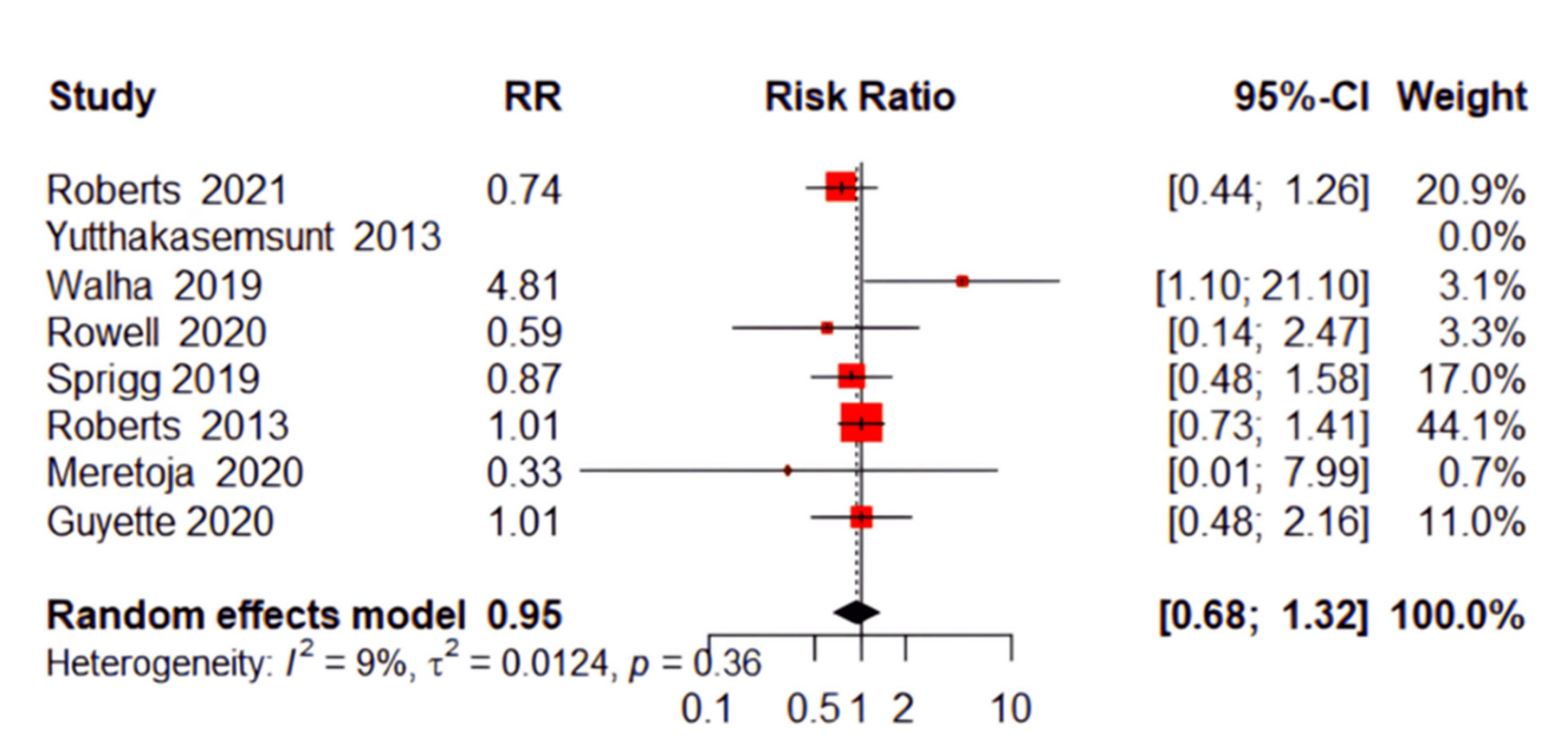
Forest plot of pulmonary embolism References [13,17,18,20-23].

Similarly, the occurrence of pulmonary embolism events did not differ significantly between the TXA and placebo groups. The pooled RR was 0.97 (95% CI: 0.78 to 1.22, P=0.82, Figure 7), indicating no protective or detrimental effect of TXA on PE risk. The heterogeneity among these studies was low (P=0.40; I²=4%).

Myocardial Infarction (MI)

**Figure 8:**
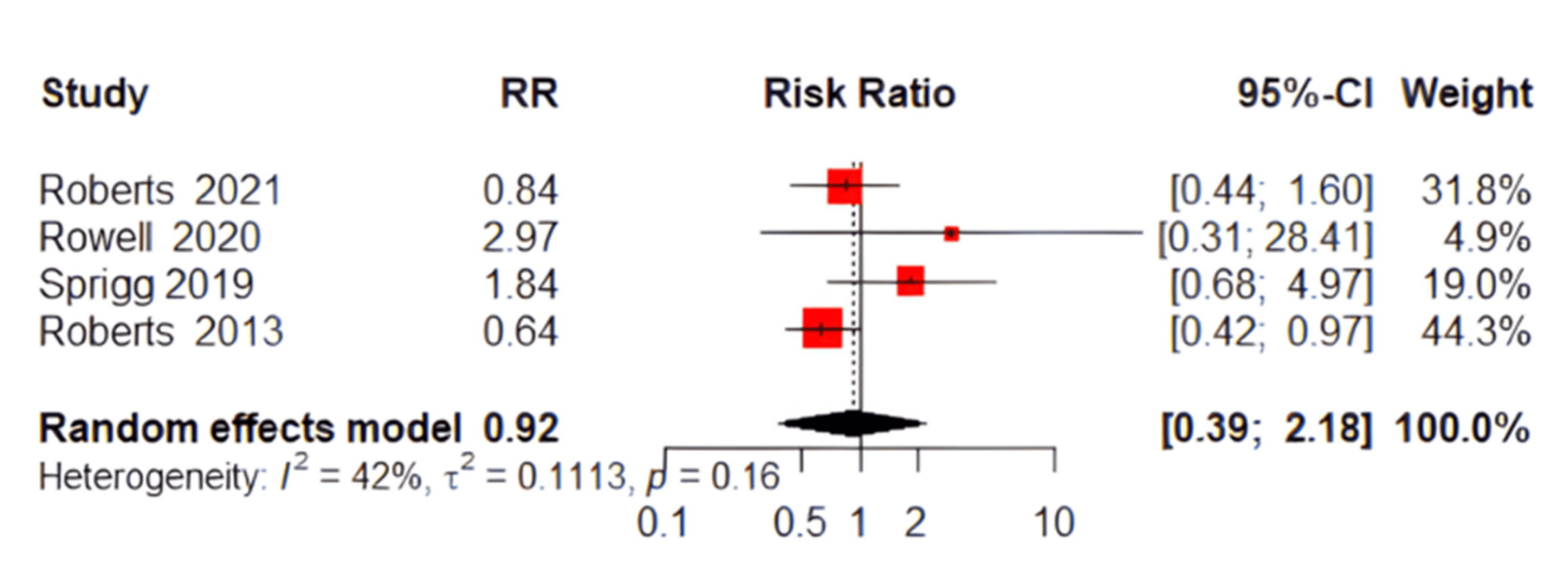
Forest plot of myocardial infarction References [17,18,20,22].

Analysis of myocardial infarction events showed no significant difference between the TXA and placebo groups. The pooled RR was 0.92 (95% CI: 0.39 to 2.18, P=0.67, Figure 8), reflecting no meaningful impact of TXA on MI incidence. The heterogeneity among the studies was moderate (P=0.16; I²=42%).

Stroke

**Figure 9:**
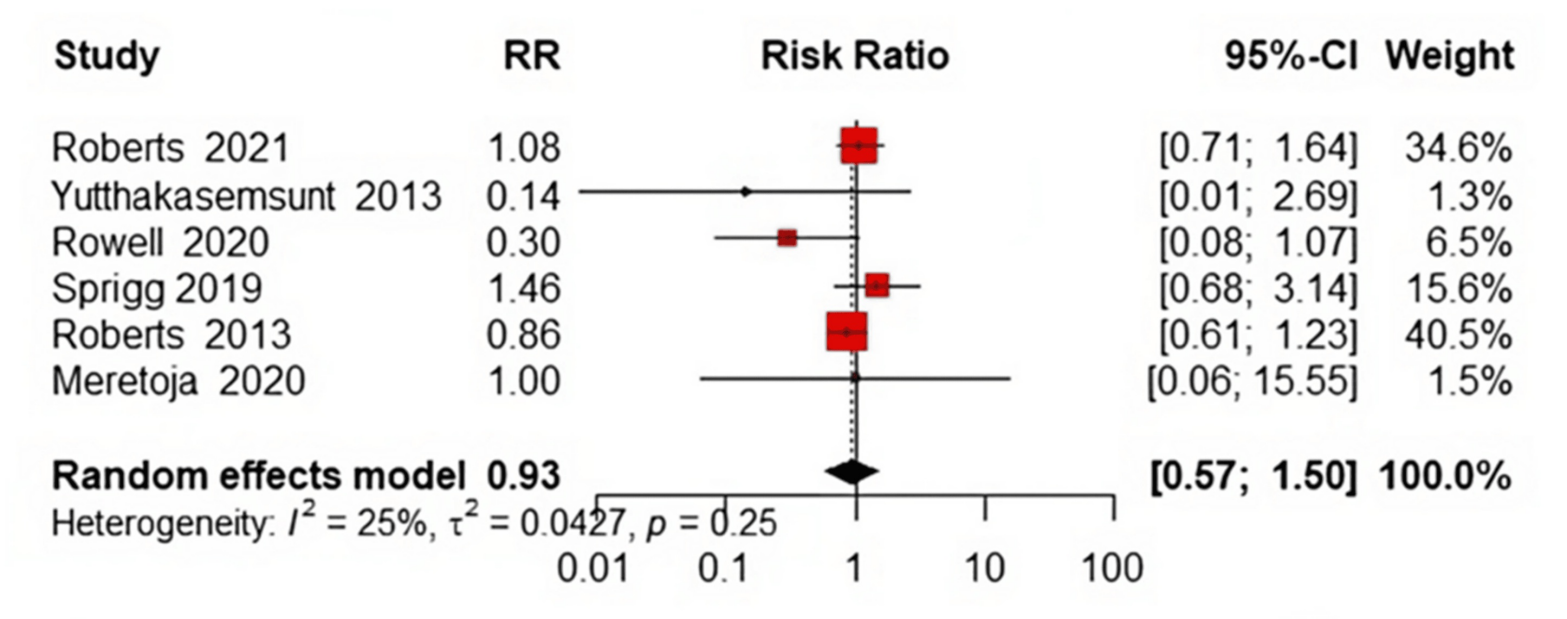
Forest plot of stroke References [17-23].

The risk of stroke was evaluated, and results indicated no significant difference between the TXA and placebo groups. The pooled RR was 0.93 (95% CI: 0.57 to 1.50, P=0.67, Figure 9), suggesting that TXA does not significantly affect stroke risk. The studies were relatively homogeneous in this analysis (P=0.25; I²=25%).

Discussion

This systematic review and meta-analysis provides a comprehensive evaluation of the efficacy and safety of tranexamic acid (TXA) in patients with traumatic brain injury (TBI). Our findings demonstrate a significant reduction in overall mortality (RR 0.95, 95% CI (0.90 to 0.99), P=0.002), decreased hemorrhagic expansion, and no notable statistically significant increase in adverse events, including thromboembolic complications. These results reinforce TXA's potential role as a beneficial therapeutic in acute TBI management, though its impact on long-term functional outcomes remains to be fully elucidated.

Efficacy of TXA on Mortality and Hemorrhage Control

The significant reduction in mortality observed in our meta-analysis aligns with the results of major studies such as CRASH-3, which emphasized the importance of timely TXA administration, particularly within 3 hours of injury. By limiting ongoing hemorrhage and reducing the likelihood of hematoma expansion, TXA provides a critical window for intervention in the acute phase of TBI. Our findings suggest that TXA's antifibrinolytic properties directly limit bleeding, thereby reducing secondary complications that typically worsen patient outcomes [26].

However, despite the clear reduction in hemorrhage volume, we observed no statistically significant impact on the need for neurosurgical intervention or functional recovery as measured by disability scales. This discrepancy highlights the complex nature of TBI pathophysiology and suggests that while TXA is effective in controlling early-stage bleeding, its role in altering the course of severe brain injuries requiring surgical management may be limited. The lack of effect on functional outcomes could be due to several factors, including the heterogeneity of TBI severity in study populations, variations in rehabilitation protocols, and the relatively short follow-up periods in many studies [27].

Safety Profile of TXA

A key finding of our analysis is the favorable safety profile of TXA, which aligns with other major studies. Neither our results nor those from previous meta-analyses identified statistically significant increases in thromboembolic events, including deep vein thrombosis, pulmonary embolism, or stroke. This is a crucial observation, given the inherent risk of prothrombotic complications in trauma patients.

Our data indicate that TXA does not increase the risk of adverse vascular events, even when used in the context of severe traumatic injuries. The absence of significant thromboembolic complications supports the continued use of TXA in emergency trauma settings, provided that it is administered within the optimal time frame to minimize risks. However, as TBI patients often present with complex comorbidities, further research is necessary to evaluate TXA's safety in patients with pre-existing cardiovascular risks, coagulopathies, or other conditions that might influence its risk-benefit profile.

Mechanism of Action and Timing of TXA

The effectiveness of TXA in limiting hematoma expansion underscores its antifibrinolytic mechanism of action. By inhibiting the breakdown of clots, TXA helps to stabilize intracranial hemorrhage and prevent secondary bleeding that can exacerbate brain injury. This effect is particularly important in the early stages following trauma, where ongoing hemorrhage can significantly worsen neurological outcomes.

Our findings, along with evidence from other studies, emphasize that early intervention is critical. Administering TXA within Three hours of injury is associated with greater reductions in hemorrhagic volume and better overall survival outcomes [9]. Delayed administration appears to diminish its therapeutic potential, suggesting that rapid deployment in trauma settings is essential for maximizing the benefits of TXA. This time-dependent effect underscores the need for streamlined protocols and efficient pre-hospital and emergency department systems to ensure timely TXA administration [28].

Comparison with Previous Meta-Analyses

Our meta-analysis builds upon and extends the findings of several key studies published in recent years. The studies by Sarhan et al. (2024) [29], Xiong et al. (2023) [30], and Zhang and Liu (2024) [31] offer valuable insights that both align with and diverge from our findings [13-25].

Sarhan et al. (2024) [29] analyzed eight trials with 10,860 patients, finding a significant reduction in hematoma expansion but no significant effect on mortality. This contrasts with our mortality findings, possibly due to differences in study populations, TXA dosing, or timing of administration. The consistency in findings regarding neurosurgical intervention reinforces the idea that while TXA may improve survival, it may not alter the underlying severity of intracranial injuries requiring surgical management.

Xiong et al. (2023) [30] conducted a more expansive analysis of 25 RCTs, demonstrating significant reductions in hematoma growth in both TBI and other intracranial hemorrhage patients. Their detailed exploration of subarachnoid hemorrhage patients provides additional context for TXA's broader hemostatic efficacy. The consistency in safety findings across these meta-analyses further substantiates TXA's favorable risk profile in neurotrauma patients.

Zhang and Liu (2024) [31] provided a detailed overview of TXA's effects, highlighting the critical importance of early administration. Their findings on time-dependent efficacy align closely with our results [13-25] and those of major trials like CRASH-3, emphasizing that early intervention is key to maximizing TXA's benefits in reducing intracranial hemorrhage and potentially improving survival outcomes.

Strengths and limitations

This study strengthens the reliability of its findings through a comprehensive search across multiple databases and strict adherence to PRISMA guidelines. By including a large patient population from various studies and employing standardized quality assessment tools, this analysis enhances statistical power, particularly in assessing mortality reduction as a primary outcome.

Despite these strengths, this study has several limitations. The variability in TBI severity and TXA dosing protocols across studies complicates the assessment of TXA's efficacy for specific TBI subgroups. Additionally, inconsistencies in outcome measures, such as functional recovery and quality of life, limit comprehensive conclusions. Short follow-up periods in many studies hinder our ability to evaluate TXA's long-term effects. Potential publication bias may also skew results toward positive findings despite attempts to adjust for it. Finally, the predominance of studies from high-income countries limits the generalizability of our findings to diverse global settings.

Clinical implications and future directions

This meta-analysis affirms the potential of tranexamic acid (TXA) to decrease mortality rates in traumatic brain injury (TBI) patients, highlighting its value as a cost-effective option for early intervention, particularly in environments with limited access to neurosurgical care. However, it is essential to note that TXA alone does not address secondary outcomes, such as the necessity for neurosurgery, underscoring its role within a broader, multidisciplinary strategy that incorporates neurosurgical intervention and intracranial pressure management. 

To maximize the benefits of TXA, future research should focus on identifying patient subgroups that gain the most benefit, refining dosing regimens, and exploring alternative administration routes that facilitate rapid response in various settings. Studies should also evaluate TXA's long-term impacts on functional outcomes, its cost-effectiveness in low-resource environments, and potential combinations with other neuroprotective therapies. Further exploration of TXA's molecular impacts on TBI could enhance treatment protocols and inspire the development of improved antifibrinolytic therapies. Addressing these research priorities could strengthen TXA's role in TBI management, ultimately leading to better patient outcomes globally.

## Conclusions

This systematic review and meta-analysis demonstrate that TXA reduces mortality in traumatic brain injury (TBI) patients without raising thromboembolic risks. Although it has a limited impact on secondary outcomes like neurosurgery needs, TXA remains beneficial for acute TBI management, especially in urgent settings. These findings support integrating TXA into TBI treatment protocols. Future research should refine patient selection, optimize dosing, and examine long-term effects to enhance survival and recovery outcomes for TBI patients globally.
